# Institutional profile: Community for Open Antimicrobial Drug Discovery – crowdsourcing new antibiotics and antifungals

**DOI:** 10.4155/fsoa-2016-0093

**Published:** 2017-03-24

**Authors:** Mathilde R Desselle, Ruth Neale, Karl A Hansford, Johannes Zuegg, Alysha G Elliott, Matthew A Cooper, Mark AT Blaskovich

**Affiliations:** 1 Community for Open Antimicrobial Drug Discovery, Institute for Molecular Bioscience, The University of Queensland, Brisbane QLD 4072, Australia

**Keywords:** antibiotics, antimicrobial activity, antimicrobial resistance, chemical diversity, medicinal chemistry, open-access drug discovery, organic chemistry, synthetic chemistry

## Abstract

The Community for Open Antimicrobial Drug Discovery (CO-ADD) is a not-for-profit, collaborative approach to discovering new antibiotics. We access novel chemical diversity from academic synthetic chemists, who collectively possess millions of untested compounds with chemical diversity that lie outside commercial collections. We perform high-throughput antimicrobial screening of pure compounds derived from both synthetic and natural sources free of charge. The resulting data can be used by participants for publication, patenting and development purposes, and is fed back into the research community through an open-access database after a 2-year period during which information is kept confidential to the provider. CO-ADD is fundamentally asking two questions: can the community work together to address the global threat of antimicrobial resistance; and are there as yet undiscovered, novel antimicrobial compounds already present within our diverse global chemistry community?

One critical bottleneck in the discovery of new antibiotics is the limited synthetic chemical diversity screened for antimicrobial activity, and the accessibility and suitability of commercial screening initiatives [1], where infectious diseases have traditionally had a low priority compared with metabolic and chronic diseases.

The Community for Open Antimicrobial Drug Discovery (CO-ADD) was launched in February 2015 at The University of Queensland’s Institute for Molecular Bioscience in Brisbane, Australia. The program is supported by a Wellcome Trust Strategic Award and The University of Queensland, with a team of 15 chemists, microbiologists and outreach professionals actively targeting chemists in academia and research organizations. These chemists have compounds ‘sitting on shelves’ that were not necessarily designed as antibiotics, which would not otherwise be screened for antimicrobial activity. CO-ADD [2–4] tests submitted compounds against a panel of some of the most serious microbial threats to human health, including *Escherichia coli*, *Klebsiella pneumoniae*, *Acinetobacter baumannii*, *Pseudomonas aeruginosa* and methicillin-resistant *Staphylococcus aureus*, as well as the fungal pathogens *Cryptococcus neoformans* and *Candida albicans*. Academic compounds are screened at no cost to the submitter through a cascade of assays as illustrated in Figure 1 and outlined below.

- Primary screening: All submitted compounds tested at a single concentration (32 µg/ml).
- Hit-confirmation: All actives from the primary screen tested in dose response antimicrobial assays to confirm their activity, with additional assays to profile for adverse effects (cytotoxicity and red blood cell hemolysis), nonspecific effects (critical micelle concentration) and purity (LC-MS analysis).
- Hit-validation: Confirmed hits further screened against a broader panel of bacteria and fungi including multidrug-resistant clinical isolates, in the presence of serum and profiled for preliminary drug-like properties, including microsome and plasma stability and protein binding.

**Figure 1.**
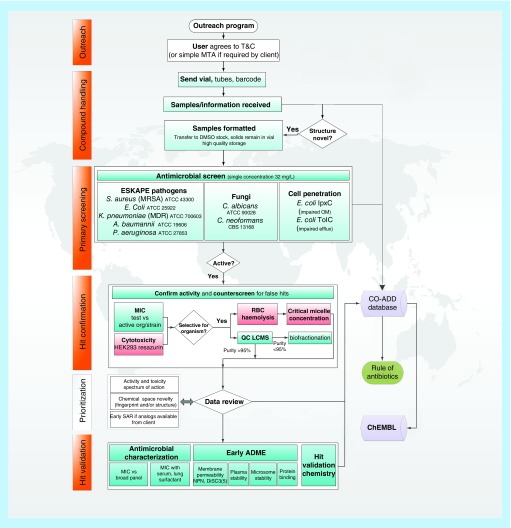
**The Community for Open Antimicrobial Drug Discovery antimicrobial screening workflow.** ADME: Absorption, distribution, metabolism, excretion; CO-ADD: Community for Open Antimicrobial Drug Discovery; LCMS: Liquid chromatography mass spectrometry; MTA: Material transfer agreement; OM: Outer membrane; QC: Quality control; SAR: Structure–activity relationship; T&C: Terms and conditions. Reproduced with permission from [5].

Both positive- and negative-screening results are made publically available in an open-access database within 24 months following the completion of the primary screening process.

Since February 2015, CO-ADD has received 130,000 molecules from 34 countries (Figure 2). To date, the screening of 100,000 compounds has identified 584 compounds that pass the hit confirmation criteria. Such compounds display antimicrobial activity against at least one of the target organisms (MIC ≤16 µg/ml) and demonstrate at least some selectivity over mammalian cells, in other words, the concentration causing toxicity to mammalian cells (50% cytotoxic concentration, CC_50_) is higher than the concentration needed to inhibit microbial growth (MIC). Such compounds may represent promising starting points for antibiotic development.

**Figure 2.**
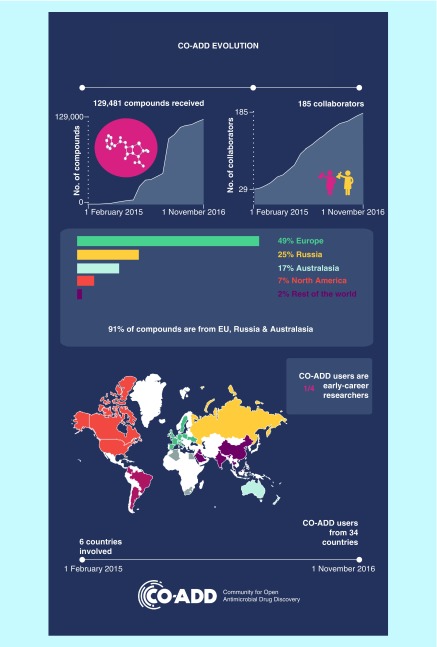
**The Community for Open Antimicrobial Drug Discovery engagement evolution.** CO-ADD: Community for Open Antimicrobial Drug Discovery.

## Community outreach

To engage chemists in academia, CO-ADD is running a number of outreach programs with the following goals:

### Development of a high visibility brand to build an international community

CO-ADD has developed a website for static information, an online compound submission form and a secure data distribution portal. Large advertising campaigns using Search Engine Optimization (Google AdWords) and social media has led to 400,000 impressions and 10,000 page views per month. CO-ADD also engages with the media on a monthly basis, and organizes scientific forums and community events. Examples include an Australian Broadcasting Corporation (ABC) TV episode of Catalyst ‘Antibiotic Resistance’ [6], an ABC TV News ‘New antibiotics needed to fight drug-resistant bacteria’ [7], an ABC Radio National ‘Future Tense’ episode on ‘Drug resistance and a coming pandemic’ [8], a ‘Superbugs at the Olympics’ evening community event [9] and a TedX talk [10].

### Creation & maintenance of an engaged network of users

A customer relationship management system has been set up to manage contacts and enquiries in a confidential manner and enable the broadcasting of mailings and monthly newsletters. The team is also presenting at conferences worldwide on a regular basis, with the initiative supported by a number of high-profile advocates (politicians, patients, researchers and clinicians) and a network of 20 international champions. As a result, CO-ADD has engaged 185 collaborators in 18 months, a substantial 26% of which are early career researchers, demonstrating that these young researchers entering the competitive research world are benefiting from the CO-ADD initiative. The free testing provides early career researchers with research data that they would otherwise be unlikely to obtain, potentially leading to additional publications, novel avenues of research and new funding opportunities.

For management of sample submissions and data reports, CO-ADD has established a secure online user portal, where participants can view their projects, upload new compound submissions and access screening reports. The time from compound submission to release of hit confirmation data to the participant is generally 8 weeks, which is a significant improvement over the lag times often encountered with bulk screening services or national compound collections. The rapid turnaround adds to the attractiveness of participating in CO-ADD.

### Attraction of key partnership opportunities

CO-ADD has set up a number of key partnerships with chemical societies, such as the Royal Chemistry of Society (UK) and the American Chemical Society (USA), online networks, such as MedChemNet and the Infectious Disease Hub, research bodies, such as the French National Chemical Bank, and large consortiums, such as the Innovation Medicine Initiative (Europe) and the African Network for Drug Development (Africa/United Nations).

Despite CO-ADD’s initial success in engaging the scientific community, there is still much to learn about the most effective ways to engage collaborators around the globe in an open-access initiative, especially in developing countries. Skepticism that you ‘can’t get anything for nothing’, a lack of global awareness of the Wellcome Trust organization, and requirements from institutions for comprehensive legal agreements before compound submission, are some of the obstacles that need to be overcome. By leveraging the tools traditionally used in a commercial environment such as digital advertising and service-type data management software, CO-ADD is able to reach out globally to initiate the conversation that will nurture a sustainable community approach toward tackling one of the greatest threats facing human health.

## Screening outputs

### CO-ADD hit rate

In a pilot study, CO-ADD screened 140,000 compounds from ‘drug-like’ commercial libraries. A comparison of the hit rates between these commercial libraries and the academic collections screened thereafter revealed a 25-fold higher hit rate for the academic compounds, supporting the hypothesis that academic compound collections may be better suited to finding starting points for antibiotic development (Table 1).

**Table 1.** **Community for Open Antimicrobial Drug Discovery hit rates as of November 2016.**

**Hit rate (confirmed hits with selectivity over mammalian cell cytotoxicity)**	**G+ve**	**G-ve**	**Fungi**
CO-ADD academic hit rate	0.66%	0.21%	0.98%
Commercial hit rate	0.03%	0.008%	ND

CO-ADD: Community for Open Antimicrobial Drug Discovery; ND: Not determined.

### Case study

A partnership between CO-ADD and the ND Zelinsky Institute of Organic Chemistry of the National Academy of Sciences in Russia has been established to screen 150,000 compounds that were originally collected in the context of an NMR project. The collaborator agreed to provide the compounds in a transparent manner (i.e., structures disclosed) via monthly shipments of plated compounds. A collection of 35,520 compounds has already been shipped to CO-ADD, with a screened subset producing ten hits identified during hit-confirmation. The collaborator has provided supporting cytotoxicity and activity data, as well as analogs where available. Validated hits will be taken further into drug optimization programs.

### Open/big data

Open access is changing the global science publishing world. It enables researchers to carry out collaborative research on a global scale and can transform publications and data into a far more powerful resource for research. CO-ADD is part of this new paradigm, and is building an open-access repository for antibiotic drug discovery with both positive and negative results, available for all academics to access and analyze. Importantly, the database contains datasets derived from standardized assays, in other words, screening results from a single screening site under a standard set of conditions. In the context of antimicrobial screening, the value of user generated data relies heavily on the quality and consistency of the data being generated, as assays can be performed under many different conditions using different bacterial strains, growth conditions and end point readouts. The availability of a global screening data set, measured under identical experimental conditions, using readily accessible pathogen strains, is highly valuable to the drug discovery community. CO-ADD captures all screening results within an in-house-built data repository, currently containing 2.1 million individual data points. This raw data, together with experimental protocols and chemical structures of all compounds, will be made publicly available 24 months following the completion of the screening process, unless more time is required by the chemist to publish or commercialize their project. In an otherwise competitive drug discovery arena where big data is often confidential, CO-ADD aims to provide an open-access database of antimicrobial screening knowledge. Such an endeavor will give the global antimicrobial research community a well needed boost, while in the process avoiding the unnecessary duplication of screening efforts.

### Hit development options

CO-ADD provides researchers with a complete data package to assess the suitability of a candidate for progression into a hit-to-lead or lead optimization program. A compound series that has progressed through hit validation will have been assessed for antimicrobial activity against a range of standard and drug-resistant isolates, including both ATCC reference [11] and clinically acquired strains. Compounds will have been re-synthesized alongside several analogs to confirm that there is scope for further hit optimization, and profiled to gauge initial drug-like properties (in particular stability under biological conditions). Under CO-ADD’s terms of agreement, participants retain all rights to this compendium of data, and are free to patent their discovery and develop their series independently. A significant proportion of compounds submitted to CO-ADD are from researchers not involved in antibiotic discovery. Such groups may lack the appropriate resources, expertise or collaborations to advance their compounds further. Participants can leverage CO-ADD’s expertise in antibiotic discovery by partnering with CO-ADD to generate additional data that may be required for entry into the new antibiotic development initiatives, or obtain guidance from CO-ADD on potential pathways for further development. Indeed, CO-ADD encourages the participating group to engage in the development and optimization of their compound, thereby seeding new antimicrobial research in academia.

## Future plans

A number of government action plans on combating drug-resistant bacteria have recently been published [12–14]. Public–private partnerships for the development of new antibiotics were announced in 2014 by the European Union IMI ENABLE [15] consortium (€100 million), and in August 2016 by the CARB-X Initiative. The latter is led by Boston University Law School and the Department of Health and Human Services in the USA, and the Wellcome Trust and the new AMR Centre in the UK [16], leveraging US$250 million. The Broad Institute announced a Collaborative Hub for Early Antibiotic Discovery [17] supporting CARB-X. DNDi also launched its Global Antibiotic Research Development Partnership (GARDP) [18] with €2 million seed funding.

These initiatives highlight the need to develop new antibiotics. However, public–private partnerships are looking for compounds that are already at an advanced hit-to-lead, optimized lead or even candidate drug stage, while the major issue with the antibiotic discovery pipeline is the lack of truly novel chemotypes that would be eligible to access these initiatives. There are very few early-stage Phase I candidates currently known to be in testing. About 40 new antibiotic candidates are currently in the development pipeline, mostly in Phase II and Phase III [19], with only 10–15 having completely novel chemical scaffolds [20].

CO-ADD is assembling the world’s largest, most diverse collection of compounds, with over 300,000 additional compounds identified as available in addition to the 130,000 already received. We have seen a very rapid uptake in participation compared with other initiatives, such as national compound collections or public–private screening programs (e.g., the Eli Lilly Open Innovation Drug Discovery initiative). This rapid uptake may be attributed to:

- The specific focus on an urgent unmet medical need with concomitant global recognition of the consequences of antimicrobial resistance;
- The rapid turnaround of screening results (e.g., results provided to an early career researcher submitter in time to be incorporated into their thesis or papers, rather than at some nebulous time in the future);
- The low barriers to participation, with minimal paperwork and no participation criteria other than being in an academic institution.

The diversity contained within CO-ADD’s collection also has applications beyond the current bacterial and fungal focus. With continued funding, CO-ADD is looking to expand its campaign to include screening for compounds active against other key pathogenic diseases, including tuberculosis, dengue and parasitic diseases such as malaria, Chagas’ disease and leishmaniasis. We also hope to extend the open-access concept to include a synthesis component, modeled on the Open Source Malaria project [21].

In summary, CO-ADD is rebooting the antibiotic discovery pipeline by tapping into untested chemical diversity held by academic chemists. The early adopter initial uptake has been gratifyingly positive and screening results are promising. We hope that in the long-term CO-ADD will help researchers generate new antibiotics and antifungals that reach the clinic.
